# Attitudes to physical healthcare in severe mental illness; a patient and mental health clinician qualitative interview study

**DOI:** 10.1186/s12875-020-01316-5

**Published:** 2020-11-27

**Authors:** Joseph Butler, Simone de Cassan, Phil Turner, Belinda Lennox, Gail Hayward, Margaret Glogowska

**Affiliations:** 1 Foundation Year 3 Physical Health Care, Department of Psychiatry, University of Oxford, Oxford, UK; 2 Oxford Health NHS Foundation Trust, Oxford, UK; 3 NIHR Community Healthcare MIC, Nuffield Department of Primary Care Health Sciences, University of Oxford, Oxford, UK; 4 Department of Psychiatry, University of Oxford, Oxford, UK; 5 Nuffield Department of Primary Care Health Sciences, University of Oxford, Oxford, UK

**Keywords:** Mental health, Qualitative research, Health promotion and prevention

## Abstract

**Background:**

People with severe mental illness experience physical health significantly worse than the general population. Physical health monitoring is shared between primary care and secondary mental healthcare services, though there is debate whether mental health teams should provide more physical healthcare. The views of mental health clinicians and patients with mental illness towards physical healthcare provision are unclear.

**Aims:**

To explore the attitudes of Community Mental Health Team (CMHT) clinicians and patients experiencing severe mental illness towards physical healthcare and its provision.

**Design and setting:**

Qualitative study in a CMHT setting.

**Methods:**

Interviews were carried out with CMHT clinicians and patients with severe mental illness. Data were collected using semi-structured interviews and analysed using thematic analysis.

**Results:**

There were 14 patients and 15 clinicians recruited. Patients varied in their awareness of the association between physical and mental health, but were engaged in physical health monitoring. Clinicians were aware of the importance of physical healthcare but reported barriers to provision, including lack of training, resource constraints and uncertainty in their role. There was no consensus in either group regarding how physical healthcare should be provided, with diverse attitudes expressed for why CMHTs should and shouldn’t provide more physical healthcare.

**Conclusions:**

Increasing physical healthcare provision from mental health teams requires healthcare-related barriers be addressed, but it remains unclear whether CMHT clinicians or patients believe this to be a solution.

## Introduction

The mainstay of psychiatric care in the UK is delivered by Community Mental Health Teams (CMHTs). CMHTs operate alongside General Practitioners (GPs) in the community and as a result, have joint responsibility for certain aspects of patient care, in particular the physical health of patients with severe mental illness (defined as schizophrenia, bipolar affective disorder and other psychoses) [1]. Psychotic Disorders are associated with a 15–20 year mortality gap [2–4] mediated primarily by cardiovascular comorbidity [5]. Against a background of increasing multi-morbidity, the numbers of people living with both physical and mental health conditions is rising [6, 7], which is associated with disproportionate increases in healthcare utilisation [8].

The National Institute of Clinical Excellence recommends annual physical health checks for those with severe mental illness [9]. Primarily a screening tool, it allows patients with high cardiovascular risk to be identified and receive personalised interventions. Completion of this physical health check is poor; only 32.3% of those with severe mental illness receive a full check [10]. Furthermore, it remains unclear where mental health clinicians and their patients believe responsibility for physical healthcare lies and whether this ambiguity may be a barrier to optimal patient-centred (rather than disease-centred) care. Some argue that the ‘shared care model’ creates a system where roles are blurred, responsibility is ambiguous and patients can ‘fall through the cracks’ [11]. Others argue that further specialisation leads to worse patient outcomes and inefficient functioning of the healthcare service [12].

Consensus amongst policymakers is moving towards a view that physical health monitoring should be part of mental health practice [13]. Whilst a lack of clarity in the role of the CMHT has been suggested as a barrier [14] to achieving this, there is limited data exploring patient and clinician attitudes to physical healthcare in mental illness. As part of a mixed methods evaluation of a Point of Care (POC) blood testing device in two CMHTs [15], we conjunctively interviewed both patients with severe mental illness and the clinicians caring for them. The POC device allowed clinicians to sample and record HbA1c and Lipid Panel blood tests, and independently complete a full physical health check in one sitting. The interviews aimed to explore both groups’ attitudes to the physical health check and more broadly, the role of mental healthcare in providing physical healthcare. Understanding these stakeholders’ views is necessary for improving physical healthcare for people with severe mental illness.

## Methods

### Ethical approval

Ethical Approval was prospectively provided after proportionate review by the sub-committee of Wales Research Ethics Committee 6 (Reference: 18/WA/0302).

### Eligibility and recruitment

This study forms part of a larger mixed-methods evaluation of a POC device in two community mental health teams; the Oxfordshire Early Intervention in Psychosis Service (EIS) and the South Oxfordshire Adult Mental Health Team (AMHT) who each had the ‘*Afinion 2’* POC device for 6 months between May and December 2019. Clinicians and patients were informed the device was being trialled as a service evaluation, aiming to improve delivery of the physical health check and that if successful, the device could be embedded long-term in the service. It was explained that interviews were to evaluate the device, and to explore long-term perspectives of physical health.

#### Clinician study

Clinicians who had access to Lipid and HbA1c POC testing for home visits and outpatient clinics were approached for participation by the research team, and given a study information leaflet.

#### Patient study

Patients experiencing severe mental illness are eligible for a yearly physical health check. In the context of this cohort this was limited to patients who experience psychotic symptoms as part of their condition. Patients eligible for a physical health check, who had had their care augmented by the POC device, were given study information leaflets by their Care coordinator during the clinic appointment. If they wished, they could provide contact details in order to be contacted by one of the research team.

### Data collection

All participants interviewed gave informed consent for participation in the study and for publication of the project findings and written quotations. If willing to participate, consent was taken with a researcher, either in person or telephonically. Consent was either taken when organising the interview, or immediately prior to the interview taking place. If being conducted telephonically, the consent form was read out to the participant and signed by the clinician. In these cases a paper copy of the consent form was sent to the participants.

Semi-structured interviews were conducted by JB and SdC using a topic guide to explore perceptions and experiences. Separate topic guides for Clinicians and Patients were developed, initially from the literature and the experience of our research team. The research team met after the initial interviews and then frequently to discuss the suitability of the topic guide, and adapted it iteratively as new topics evolved and emerged.

### Data analysis

Audio-recordings were independently transcribed verbatim and analysed thematically. Patient and Clinician data were analysed in parallel using the constant comparative method [16]. Researchers read and familiarised themselves with the transcripts, noting and recording initial themes, then conducted systematic and detailed open coding using QSR NVivo 11. The coding framework was derived from the topic guide and refined after initial double coding of transcripts by SdC and JB as well as discussions amongst the whole research team. Subsequent coding and analyses were completed by SdC and JB, with further group discussion to resolve differences and combine or remove codes where appropriate. The research team took an iterative stance, taking forward early analysis into ongoing data collection allowing for the inclusion of emerging categories from the data ensuring themes and concepts were grounded in the data.

## Results

Fourteen patients were interviewed, ten male and four female. The average age was 33 (19–66). All came from the EIS caseload. Fifteen clinicians were interviewed, seven male and eight female. Four clinicians came from the CMHT, 11 came from the EIS. Clinician backgrounds included Psychiatry (*n* = 3), nursing (*n* = 6), other allied health care professionals (*n* = 6). Our analysis of transcripts revealed a number of themes which are summarised in Table 1.

**Table 1:** Table of Themes

Theme	Sub-theme
Awareness of Physical Health	• Clinician awareness of patient physical health
	• Patient awareness of their own physical health
Why Patients engage with the Physical Health Check	
Barriers to Clinician engagement with the Physical Health Check	• Clinician background and training
	• Clinician uncertainty in role
	• Practicalities and resource constraints
Uncertainty in how physical healthcare should be provided	• Clinician perspectives
	• Patient perspectives

### Awareness of physical health

#### Clinician awareness of patient physical health

All clinicians expressed an awareness of the effect severe mental illness had on the physical health of their patients, and a subsequent desire to improve physical healthcare:

*“I think* [Physical Health is] *really important because of the effect on life expectancy.”* (C009, Medical, EIS)

There was not a consensus on the magnitude or trend of physical health inequality. Some believed the problem was worsening due to an aging population on long-term atypical antipsychotic medication. Others felt the opposite as the effects of improving healthcare hadn’t become apparent yet:

*“I think we’ve got a real issue coming up with the generation of people who are on… particularly on atypical anti-psychotics as they get older.”* (C012, Medical, AMHT)

*“I appreciate the statistics suggest we’re not on top of it* [Physical Health]*, but I think that’s because the statistics don’t tell the longer story of changes that have happened because people who are living longer haven’t died yet.”* (C011, Nursing, EIS)

Clinicians were aware that their patients knew the effects of medication on their physical health. This situation was outlined by a patient recalling an experience from an inpatient ward that was subsequently relayed to their care co-ordinator:

*“…his first experience when he got admitted to the wards was somebody saying to him, ‘This must be your first time* [episode of psychosis]*,’ and he says, ‘Why do you say that?’ He said, ‘Because you’re not overweight.’ The patients know it themselves.”* (C008, Social Work, EIS)

#### Patient awareness of their own physical health

Awareness of physical health varied across patients. Most stated their physical health was important, but were unable to elaborate further, despite encouragement by the interviewer. A number did have clear convictions of what contributed to their *cardiovascular* health, including knowledge of smoking, drugs, exercise, diet and family history:

*“No, I don’t drink, I don’t smoke, I don’t take drugs and I am very active. I’m partially vegetarian… my diet’s spot on. So, yeh I mean I’ve joined a gym but I haven’t actually been yet…”* (P004)

“*From just sort of lifestyle things and family history, I don’t think, you know,* [I’m] *more predisposed to things like diabetes than most people”* (P003)

### Why patients engage with the physical health check

Patients did not generally mention the association between severe mental illness and poor physical health as a rationale for the health check; most subsequently took part in the physical health check as it was offered, rather than because they thought it would personally benefit:

*“I’m not personally that worried, but because I was here and it was, you know, it’s part of the service I was quite happy to do [*the physical health check]. *It’s good to find out the results but I wouldn’t like to go out my way to go find out about it.”* (P002)

Some were more proactive, their motivations for engaging with the check reflecting underlying attitudes towards their own physical health and the value of the check as a screening tool:

*“Because my Dad’s got Diabetes… I thought there was a risk that I could have it... But the good thing about having that like physical health check is to make sure if I've got it or not.”* (P0010)

Others were motivated by their knowledge of cardiovascular risk, and the physical health check’s role as a way of determining that risk:

“*I think everyone should know their levels really. And like, you know, maybe be able to detect earlier if they’re at risk or not, so I think it’s quite important that people know really, yeh.”* (P009)

A minority were aware of the associated physical health risks with severe mental illness and conceptualised this as a rationale for the physical health check:

“*…there’s the higher risk of certain health issues, if you have a mental health issue, so they just want to keep a record and like to diagnose things early. And so, it was testing for cholesterol and like the chance of you getting diabetes or heart disease”* (P006)

There were other reasons for engaging in the physical health check. Some expressed security, knowing that their physical health was being monitored:

*“Yeh, it makes me feel like more comfortable and like my health is being checked.”* (P008)

Whilst others expressed curiosity:

*“I was very intrigued to find out what my levels were actually because I’d never had that kind of test done before.”* (P009)

### Barriers to clinician engagement with the physical health check

#### Clinician uncertainty in responsibility

Clinicians were uncertain of the role of the mental health clinician when it came to responsibility for the physical health check:

*“I think the physical health checks were quite haphazard. Some people felt it was their responsibility; some didn’t.”* (C006, Allied Health, EIS)

*“There can be differences of opinion about who should be doing the testing.”* (C012, Medical, AMHT)

Differing expectations across different mental healthcare teams could make clarifying responsibility confusing; illustrated by a psychiatrist reflecting on the differences between their time in inpatient and community teams:

*“I don’t know. I guess that… I mean its difficult because I guess my experience of being on the inpatient wards you know it’s very much been my responsibility to* [prescribe physical health medication]*, but I feel like the GP needs to be aware* [in community psychiatry]*… so there’s no kind of error or overlap. But I don’t know if it should be the case.”* (C014, Medical, EIS)

#### Clinician background and training

Clinicians’ professional and training background affected how they saw their role. Clinicians with physical health training, such as medics and nurses, tended to advocate a more proactive physical healthcare role:

*“I think we should be doing more about chasing it up and asking the clients…What is the plan? And if you don’t think the plan is concrete enough, call the GP and talk to them and say… What are you doing about it?’”* (C013, Nursing, EIS)

Whilst those with less of a physical health background, such as social workers, took a less proactive stance towards physical healthcare:

*“Unlike colleagues here that would have a health background, particularly CPNs nursing, I’m a social worker, so we are obviously not as skilled in things to do with physical health as our colleagues are… If we notice anything about those blood samples that would put it in an area where there should be some concern, rather than deal with that ourselves we should then escalate that to a designated member of staff”* (C007, Allied Health, EIS)

#### Practicalities and resource constraints

If blood tests were required, patients had to attend their GP practice for phlebotomy. Frustration was expressed at how this arrangement often did not work for clinicians or patients:

*“We were relying on the clients to go to the GP with the* [test request form] *and that very very rarely happened.”* (C006, Allied Health, EIS)

*“If the client is struggling or… lack of transportation. They probably don’t walk; they don’t have enough money to go out and properly visit their GP… And the environment as well – some of them live in rural areas and getting… accessing their GP can be quite difficult.”* (C013, Nursing, EIS)

Clinicians might want to take on more physical health check responsibility, but reported that lack of resource could be barriers to this:

*“If we had more resources then I could certainly see a case for us doing it in-house if we had the equipment and if we had the, you know, the extra staff to do it.”* (C012, Medical, AMHT)

*“You’ve got so many other competing priorities… It’s about managing their workload, you know… and physical health is often the one that slips off.”* (C009, Medical, EIS)

### Uncertainty in how physical healthcare should be provided

#### Clinician perspectives

Many clinicians believed that physical healthcare and mental healthcare should become more entwined. Justification included an opportunity to provide a more efficient service:

*“…taking on a more proactive role seeing as we are often seeing these patients most, so you know, taking on the role of prescribing statins or prescribing antihypertensives or to avoid that delay in treatment or patients not going to get help.”*(C014, Medical, EIS).

Some clinicians saw it as a way of providing more holistic care:

*“I think it’s all part and parcel of the same, you know, health provision…It shouldn’t be physical health and mental health; it should be person-centred.”* (C010, Nursing, EIS)

Others felt that because a large part of the physical health burden was secondary to a mental health condition, this is something mental healthcare should take responsibility for:

*“We should be seeing their physical health is as important as their mental health in mental health services… If they’re under our care, kind of makes sense. You know, if we’re the ones doing it to them, or potentially doing it to them.”* (C009, Medical, EIS)

This was not a universal belief; one participant felt a more integrated service might reduce patient autonomy given that they were unlikely to remain under lifelong CMHT care:

*“I personally think that people need to go to the GP... I just think kind of taking control over people’s life; we take enough control for them… if they’re going to be on anti-psychotics beyond the time with us, get into the habit of looking after their physical health and being aware of what they need to be aware of.”* (C008)

Furthermore some clinicians wanted to focus more on their client’s mental health and hand over physical health workload to the GP:

*“…I don’t think, and* [GPs] *tend to agree… that it’s a good use of our time to be doing ECGs and bloods. They’re comfortable with doing them if it means we can get on more with doing mental health stuff…Our GP’s are pretty good actually”* (C012, Medical, AMHT)

As well as healthcare system factors that can affect delivery of physical health care, clinicians also reflected upon person-level factors and practicalities of this process. Some clinicians acknowledged their privileged position as often being a patient’s primary healthcare contact, and how this might make them ideally positioned to deliver physical healthcare

*“I’ve got that relationship with them anyway; hopefully a trusting relationship with them.* [Physical healthcare] *might be a bit more meaningful to them and stick a bit more.”* (C006, Allied Health, EIS)

Patients’ expectations of their clinician worked both to promote and demote a clinician’s role in providing physical health:

*“Nine times out of ten they’d come to me and they’d say, ‘Oh I’ve got so and so, could it be the medication causing it?’… Quite often people automatically make that jump to it’s a side effect of medication, but if I feel its not, I do encourage them to go and access their GP.”* (C006, Allied Health, EIS)

*“It would be nice to feel that they could share other feelings about their physical health as well, but I don’t think that’s what they expect us to be able to comment on, and I don’t think that’s something that ordinarily they would necessarily volunteer, unless it was sought from them.”* (C007, Social Work, EIS)

#### Patient perspectives

The confusion over who should be responsible for physical healthcare, between Primary Care and the CMHT became apparent in the wide range of opinions expressed. Some patients advocated a clear split, with Primary Care dealing with physical health and the CMHT for mental health:

*“I don’t see that I would contact my co-ordinator for my physical health. I think that’s more the mental side of things. The GP would make more sense for the first port of call…”* (P014)

There were others who felt the converse, citing their existing therapeutic relationship with mental healthcare as a reason to seek physical healthcare from the mental health clinician:

“Interviewer: And if you were unwell from a physical point of view… where do you think you’d turn?”

*“I’d turn to the mental health team… because I think they understand me more”* (P013)

Most patients appreciated an overlap of physical healthcare between the CMHT and GP and expressed difficulty in knowing which team is most appropriate for the job:

*“You know if I have muscle pains or whatnot I will go to the GP for that and that’s very clear, but with stuff like… fatigue and potential* [illness], *I’m not sure… I’m doing that fifty fifty with the GP and* [Clinician’s name], s*o, there’s, yeh, hard to draw the line sometimes”* (P003)

Drawing “*the line”* became apparent in a number of patient’s stories. One reported feeling comfortable discussing physical health issues with their care coordinator:

*“If I had been at risk of diabetes or had significantly more bad cholesterol, I’m sure* [the clinician] *would have gone down that route and we would have had a much longer wider discussion about, you know, my health problems and what could be done to solve these”* (P008)

But others lamented there weren’t more physical health interventions from the Mental Health Team:

*“I do think that’s probably a shame that there’s not more access about your medical aspects from the* [mental health team]. *But I suppose they think it’s more important to go to your GP for your medical aspects”* (P012)

Despite the benefits of exercise and diet on mental health, these were seen as a ‘physical health’ intervention, and it was commented that these interventions weren’t as well provided by the mental healthcare team:

*“Psychological help and help with medications and therapy … they do that very well, but exercise seems to have just sort’ve been… so, exercise and diet, although the emphasis was placed on them I wasn’t given really any information other than regular exercising; good diet .”* (P003)

## Discussion

### Summary

Patients were motivated to engage with the physical health check, but their awareness more broadly of physical health varied. Few articulated the link between mental illness and physical health comorbidity. There was no consensus who they thought was best placed to address their physical health. They did cite their existing therapeutic relationship as a reason for an increased physical health provision by mental health services but acknowledged that CMHTs weren’t necessarily equipped to deal with physical symptoms.

Clinicians identified that whilst physical healthcare was important, there were barriers such as resource constraints, diverse professional backgrounds, unclear role expectations and pre-existing relationships with GPs that prevented clinicians taking a more active role. Like patients, clinicians varied on how much of a role in physical healthcare they thought the mental health team should take.

### Strengths and limitations

This is the first UK qualitative study to explore both CMHT clinician and their patients’ attitudes to physical health and the role of the mental health service in delivering physical healthcare. The concurrent implementation of POC testing challenged traditional healthcare pathways and allowed clinicians to reflect on their role. Interviewed clinicians came from a range of professional backgrounds, seniorities and from teams which did and did not readily implement POC into their practice.

Patients were having their routine physical health check performed entirely by their care coordinator for the first time, and similarly reflected on the roles of their care providers. Severe mental illness causes symptoms which may affect patients’ ability to express themselves fully in interviews. All patients interviewed were recruited from the EIS caseload, and were within 3 years of the initial onset of psychosis. Different attitudes may be expressed in those who have longer lived experience of severe mental illness, and in those who refused or were not offered POC testing.

The EIS cohort however, is likely to be where physical health interventions are maximally beneficial; evidence suggests that metabolic and weight disturbance is maximal in the first year of severe mental illness [17] and disturbance is more severe in those who are younger [18]. Therefore, understanding the views of this cohort and those involved in their care may be invaluable in addressing larger physical health inequality in mental illness.

### Comparison with existing literature

Evidence of mental health clinicians’ attitudes are scarce. In a 2016 UK study, inpatient psychiatric nurses felt physical healthcare should be part of their role, but that a lack of medical equipment was a barrier to delivery [19]. In the CMHT setting, clinicians in our study reported less certainty for physical health responsibility but similar resource and training constraints. The importance of coordination with Primary Care in delivering effective physical healthcare has been previously reported by patients, carers and clinicians [20].

Qualitative research in those experiencing severe mental illness show a recognition of the importance of physical health, particularly an awareness of the effects of weight gain and exercise [21]. Patients report increased satisfaction in their physical health following GP appointments [22], but report high rates of stigma, and “not being heard” when voicing physical symptoms [23]. The majority of patients from our sample were aware of how to optimise their physical health and reported feeling more secure after a physical health check.

### Implications for research and/or practice

Physical health in severe mental illness is poorly served by the current model of shared care between primary and secondary health care services. Outcomes are worse, care is fragmented and patients get ‘left behind’ despite population improvements in health [13]. It is possible these inequalities may exacerbate as a consequence of the COVID pandemic [24]. Increasing integration between physical and mental healthcare is a potential solution, but our findings show that resource constraints and uncertainty across clinicians and patients are barriers to this.

In the context of rising multimorbidity [6, 7], rising demand [25–27] and rising costs of healthcare [28], it becomes even more important to ensure that effective patient care is delivered. Improving physical healthcare in severe mental illness requires policy change, and should be partly informed by qualitative research at the interface between primary care, mental healthcare and patients with mental illness.

## Data Availability

Study data are transcripts of interviews containing identifiable information. Data is not publically available due to concerns participant privacy may be compromised. Anonymised and de-identified data may be requested from corresponding author.
